# Psychometric validation of the Weiss Functional Impairment Rating Scale-Parent Report Form in children and adolescents with attention-deficit/hyperactivity disorder

**DOI:** 10.1186/s12955-015-0379-1

**Published:** 2015-11-17

**Authors:** Kavita Gajria, Mark Kosinski, Vanja Sikirica, Michael Huss, Elayne Livote, Kathleen Reilly, Ralf W. Dittmann, M. Haim Erder

**Affiliations:** Shire, 725 Chesterbrook Boulevard, Wayne, PA 19087 USA; QualityMetric Incorporated, 24 Albion Road, Building 400, Lincoln, RI 02865 USA; Klinik für Kinder- und Jugendpsychiatrie und -psychotherapie, Universitätsmedizin der Johannes Gutenberg-University Mainz, Langenbeckstr. 1, 55131 Mainz, Germany; Paediatric Psychopharmacology, Department of Child and Adolescent Psychiatry and Psychotherapy, Central Institute of Mental Health, Medical Faculty Mannheim, University of Heidelberg, D-68072 Mannheim, Germany

**Keywords:** Weiss Functional Impairment Rating Scale-Parent Report Form, Attention-deficit/hyperactivity disorder, Reliability, Validity, Responsiveness

## Abstract

**Background:**

Measurement properties of the Weiss Functional Impairment Rating Scale-Parent Report Form (WFIRS-P), which assesses attention-deficit/hyperactivity disorder (ADHD)-related functional impairment in children/adolescents (6–17 years), were examined.

**Methods:**

Data from seven randomized, controlled trials were pooled. Analyses were conducted in two random half-samples. WFIRS-P conceptual framework was evaluated using confirmatory factor analyses (CFA). Reliability was estimated using internal consistency (Cronbach’s alpha) and test–retest reliability methods. Convergent validity was assessed using correlations between WFIRS-P domain scores and the ADHD-RS-IV and Clinical Global Impression–Severity (CGI–S) scales. Responsiveness was tested by comparing mean changes in WFIRS-P domain scores between responders and non-responders based on clinical criteria.

**Results:**

CFA adequately confirmed the item-to-scale relationships defined in the WFIRS-P conceptual framework. Cronbach’s alpha coefficient exceeded 0.7 for all domains and test–retest reliability exceeded 0.7 for all but Risky Activities. With few exceptions, WFIRS-P domains correlated significantly (*p <* 0.05) with ADHD-RS-IV Total, Inattention and Hyperactivity-Impulsivity scores and CGI–S at baseline and follow-up in both random half-samples. Mean changes in WFIRS-P domain scores differed significantly between responder and non-responder groups in the expected direction (*p <* 0.001).

**Conclusions:**

Study results support the reliability, validity and responsiveness of the WFIRS-P. Findings were replicated between two random samples, further demonstrating the robustness of results.

**Electronic supplementary material:**

The online version of this article (doi:10.1186/s12955-015-0379-1) contains supplementary material, which is available to authorized users.

## Background

Attention-deficit/hyperactivity disorder (ADHD) is one of the most common psychiatric disorders among children and adolescents aged <18 years, with worldwide prevalence estimated at a little more than 5 % [1]. It is a neurobehavioural disorder characterized by inattention, impulsivity, hyperactivity and deficits in executive function (initiate, plan and organize, set goals, solve problems, regulate emotions and monitor behaviour). As in many other psychiatric disorders, ADHD symptoms are a necessary but not sufficient condition of diagnosis. Diagnostic criteria are met only if these symptoms cause substantial impact in psychosocial functioning [2]. A diagnosis of ADHD [3] therefore implies taking into account the assessment of self-esteem, learning delays and difficulties, social skills, substance abuse and risky behaviour, disruptive behaviour, and impaired family and peer relationships [4–16]. Given the potential for functional burden that ADHD may place on children and adolescents, an improvement in functioning in these areas is of great value both to patients and their caregivers [17].

This recognition has resulted in a need to demonstrate that treatments for ADHD not only improve symptoms but also improve associated functional impairment. The inclusion of a functional outcome has been made an explicit requirement by the European Medicines Agency in their guidance document on investigational medicines for ADHD [18]. This requirement is also consistent with the broader trend of incorporating measures of functioning and well-being as outcomes in clinical trials [19]. Lastly, evidence of functional impairment is an important criteria in diagnosing ADHD [20–23].

For the purpose of evaluating functional impairment in clinical trials, it is necessary to have a reliable, valid and responsive measure of ADHD-specific functional impairment. The Weiss Functional Impairment Rating Scale-Parent Report Form (WFIRS-P) [24, 25] was developed to measure ADHD-related functional impairment and has previously been used in clinical trials of ADHD treatment for children and adolescents [5]. While the instrument has been used in previous clinical trials, there are limited published data [26] on the instrument’s measurement properties, particularly with use in clinical trials. The objective of this study was to evaluate the measurement properties of the WFIRS-P, including a confirmation of the WFIRS-P conceptual framework, and its reliability, validity and responsiveness to change.

## Methods

The data for this study came from clinical trials that were conducted in accordance with the Declaration of Helsinki, and local ethics approval in countries where trials were conducted was sought. As these analyses were retrospective secondary clinical trial data analyses, no additional approvals were sought.

### Study data

Data from a pooled sample of children and adolescent patients (*n =* 2357), aged 6–17 years, with a confirmed Statistical Manual of Mental Disorders version IV text revision (DSM-IV-TR) primary diagnosis of ADHD, who participated in one of seven Phase III randomized, double-blind, placebo-controlled trials of guanfacine hydrochloride extended-release (GXR; Intuniv, Shire US, Inc., Wayne, Pennsylvania, USA) or lisdexamfetamine dimesylate (Vyvanse, Shire US, Inc.) [27–33]. In all seven trials, the WFIRS-P was used as a measure of ADHD-related functional impairment. Analyses were conducted using data from the baseline visit and one follow-up visit, conducted approximately at the same number of days from baseline for each study (Table 1).

**Table 1:** Data sources and time points used to evaluate the measurement properties of the WFIRS-P

	Lisdexamfetamine dimesylate studies			Guanfacine hydrochloride extended-release studies			
	SPD489-317	SPD489-325	SPD489-326	SPD503-314	SPD503-312	SDP503-316	SPD503-315
(Total *N* *=* 2357)	267	336	234	314	340	338	528
Study description	Phase III, double-blind, randomized, active-controlled, parallel-group	Phase III, double-blind, placebo-controlled, randomized withdrawal	Phase III, randomized, double-blind, parallel-group, placebo- and active-controlled dose optimization	Phase III double blind, randomized, placebo-controlled, dose optimization	Phase III, double-blind, randomized, placebo-controlled, dose optimization	Phase III, randomized, double-blind, parallel-group, placebo and active reference, dose optimization	Phase III, double-blind, placebo-controlled, randomized withdrawal
Study Location	Europe, North America, Australia	Europe and U.S.	Europe	North America	U.S.	Europe, North America	Europe, North America
Inclusion Criteria	Historical or current inadequate response to MPH therapy; Informed consent; Willing to comply with all testing; Age 6–17; Meet DSM-IV-TR criteria for diagnosis of ADHD;Baseline ADHD-RS-IV total score ≥28; Blood pressure within 95th percentile for age, gender and height; functioning at age appropriate level intellectually; able to swallow capsule	Informed consent; Willing to comply with all testing; Age 6–17; Meet DSM-IV-TR criteria for diagnosis of ADHD; Baseline ADHD-RS-IV total score ≥28; Blood pressure within 95th percentile for age, gender and height; functioning at age appropriate level intellectually; able to swallow capsule	Informed consent; Willing to comply with all testing; Age 6–17; Completed minimum of 4 weeks of double-blind treatment without experiencing AEs; Satisfactory medical assessment; Blood pressure within 95th percentile for age, gender and height	Informed consent; Willing to comply with all testing; Age 6–12; Meet DSM-IV-TR criteria for diagnosis of ADHD, combined sub-type or hyperactive/impulsive sub-type; Minimum ADHD-RS-IV total score of 28; CGI-S score of ≥4 at baseline; Blood pressure within 95th percentile for age, gender and height; functioning at age appropriate level intellectually; Able to swallow capsule	Informed consent; Willing to comply with all testing; Age 13–17; Meet DSM-IV-TR criteria for diagnosis of ADHD, combined sub-type or hyperactive/ impulsive sub-type; Minimum ADHD-RS-IV total score of 32; CGI-S score of ≥4 at baseline; Blood pressure within 95th percentile for age, gender and height; functioning at age appropriate level intellectually; Able to swallow capsule	Informed consent; Willing to comply with all testing; Age 6–17; Meet DSM-IV-TR criteria for diagnosis of ADHD, combined sub-type or hyperactive/ impulsive sub-type; Minimum ADHD-RS-IV total score of 32; CGI-S score of ≥4 at baseline; Blood pressure within 95th percentile for age, gender and height; functioning at age appropriate level intellectually; Able to swallow capsule	Informed consent; Willing to comply with all testing; Age 6–17; Meet DSM-IV-TR criteria for diagnosis of ADHD, combined sub-type or hyperactive/impulsive sub-type; Minimum ADHD-RS-IV total score of 32; CGI-S score of ≥4 at baseline;Blood pressure within 95th percentile for age, gender and height; functioning at age appropriate level intellectually; Able to swallow capsule
Age range, years	6–17	6–17	6–17	6–12	13–17	6–17	6–17
Baseline	Baseline (Day 0)	Baseline (Day 0)	Baseline (Day 0)	Visit 2 (baseline)	Visit 2 (Day 0)	All ages: Visit 2 (Day 0)	Visit 2 (Day 0)
Follow-up	Visit 9/ET (Day 63)	Visit 7/ET (Day 49)	Visit 6 (Day 56)	Visit 10 (Day 56)	Visit 9 (Week 7)	Ages 6–12: Visit 12 (Week 7)	Visit 13 (Week 13)
						Ages 13–17: Visit 9 (Week 7)	

*ADHD-RS-IV* ADHD Rating Scale Version IV, *CGI–S*Clinical Global Impression–Severity, *DSM-IV-TR* Statistical Manual of Mental Disorders version IV text revision, *ET*end of treatment, *WFIRS-P* Weiss Functional Impairment Rating Scale-Parent Report Form

Data were pooled across the seven trials to increase the sample size available for each tested measurement property of the WFIRS-P. Pooling of WFIRS-P data from all seven trials provided the largest sample size for validation to date and a large enough sample to allow for a random half sample split to replicate results. As the measurement properties of an assessment instrument should hold independent of treatment, data pooling was also done across blinded treatment arms. To evaluate reproducibility of results, patients in the pooled sample were randomly split into two groups of roughly equal size (referred to as sample 1 and sample 2). Therefore, unless stated otherwise, all data analyses include four sets of results: baseline sample 1, baseline sample 2, follow-up sample 1 and follow-up sample 2.

### Study measures

#### WFIRS-P

The WFIRS-P consists of 50 questions where respondents are asked to rate their child’s functional impairment over the past month. The specific version of the WFIRS-P used across trials was Version 2 [25]. The items of the WFIRS-P are scored on a four-point Likert-type rating scale: 0 (never or not at all), 1 (sometimes or somewhat), 2 (often or much) or 3 (very often or very much) and aggregated to produce six domain scores (Family, Learning and School, Life Skills, Child’s Self-Concept, Social Activities and Risky Activities). Each of the six domains is scored omitting items with a missing or ‘not applicable’ response. Response options are assigned values from 0 to 3. According to the instructions, scores can be calculated as the number of items scored as a 2 (often or much) or 3 (very often or very much), a sum score or the mean of the non-missing items [24]. The mean of non-missing items was the scoring method used in each of the trials in this study. An overall score (summary index) is also computed from all of the WFIRS-P items. A higher score on each WFIRS-P domain and summary index indicates greater functional impairment.

#### Criterion measures

Two clinician-reported measures that were evaluated during the same visits in which the WFIRS-P was administered were used as criterion measures to evaluate the validity of the WFIRS-P. The first, the ADHD Rating Scale Version IV (ADHD-RS-IV), is an instrument used to measure the severity of ADHD symptoms based on DSM-IV criteria for the diagnosis of ADHD [34]. The instrument comprises 18 items, each rated on a four-point Likert scale. The items are scored on two subscales, each comprising nine items: inattention (odd-numbered items 1 to 17) and hyperactivity–impulsivity (even-numbered items 2 to 18) [34]. A total score is also computed from the sum of all item ratings. Higher scores indicate greater severity. The ADHD-RS-IV is widely used as a primary efficacy outcome measure in ADHD clinical trials [27, 31, 35–42].

The second criterion measure, the Clinical Global Impression (CGI) scale, is a clinician-rated global assessment of the patient’s global functioning, symptom severity and treatment response (improvement), and was developed for use in clinical trials of patients with mental disorders [43]. It comprises two single-item measures evaluating disease severity and change in patient condition since study admission. The CGI–Severity (CGI–S) scale rates how mentally ill the patient is at the time of the visit, based on the clinician’s total clinical experience with the specific population. Ratings range from 1 (normal, not at all ill) to 7 (among the most extremely ill patients). The CGI–Improvement (CGI–I) scale rates patient improvement relative to their baseline assessment symptoms. Response options range from 1 (very much improved) to 7 (very much worse), with a value of 4 corresponding to ‘no change’.

### Measurement properties

The initial step in the validation of an instrument entails the evaluation of the measurement properties involved and confirmation of the conceptual framework of the WFIRS-P as suggested by the instrument’s developer and implied in the scoring instructions for each of the WFIRS-P domains [24]. The conceptual framework explicitly defines the concepts measured by an instrument and describes the relationships between items, domains and concepts measured, and the scores produced by the instrument [44]. To confirm the conceptual framework of the WFIRS-P, confirmatory factor analysis (CFA) appropriate for categorical-level data [45] was conducted, using polychoric correlations and the weighted least square estimator with robust standard errors and mean- and variance-adjusted chi-square test statistics (WLSMV). The confirmation of the conceptual framework of the WFIRS-P is important for the purpose of supporting the recommended scoring and interpretation of scales. The goodness-of-fit of each CFA model was evaluated using the comparative fit index (CFI) [46], where the suggested cut-off for acceptable fit is CFI >0.90 [47], and the root mean square error of approximation (RMSEA), where the suggested cut-off for acceptable fit is RMSEA <0.10 [48]. Additionally, item-to-factor loadings were examined where it was expected that items would have strong loadings (>0.40) on their respective factor [49]. In confirming the conceptual framework of the WFIRS-P, two sets of CFA models were tested. First, as a base-case model, a one-factor model was tested with the data to support the scoring and interpretation of the summary index. Second, a six-factor model consistent with the conceptual framework of the WFIRS-P was tested and compared against the one-factor model. The *a priori* pre-specified hypothesis was that the six-factor model representing the conceptual framework of the WFIRS-P would show a much better fit than a one-factor model. Moreover, showing a better fit of the six-factor model over a uni-dimensional model supports the interpretation of each domain as potentially representing distinct and independent concepts of ADHD-related functional impairment.

The reliability of the WFIRS-P domain scores was tested using internal consistency and test–retest reliability methods. Internal consistency reliability evaluates the extent to which the items of a scale measure the same concept. Cronbach’s alpha [50] was computed at baseline and follow-up assessments were performed to estimate the internal consistency reliability of each WFIRS-P domain. Test–retest reliability is the degree to which repeated administration of an assessment produces similar results in a sample where no change has occurred. This was assessed by computing the intra-class correlation coefficient (ICC) between WFIRS-P domain scores at two time points based on an analysis of variance (ANOVA) model [51]. As test–retest reliability assumes that there has been no change in the concept of interest, data from clinical trials are not ideal for this purpose due to an external factor such as a pharmacological or other intervention. To minimize the effects of this potential limitation, evaluation of test–retest reliability was limited to three of the clinical studies that had a short period of time (2–3 weeks) between consecutive visits in which the WFIRS-P was administered. The sample was further limited to patients who were rated as ‘no change’ on the CGI–I scale at the second of the consecutive visits.

Spearman’s rank correlations were computed to assess the convergent and divergent validity of the WFIRS-P domains. Convergent validity is a subtype of construct validity. Convergent validity refers to the degree to which two measures of constructs that theoretically should be related, are, in fact, related [41]. Specific criterion measures used to assess the convergent validity of the WFIRS-P domains included the ADHD-RS and the CGI–S. Symptoms of ADHD are known to have an adverse impact on child functioning and well-being [52, 53]. Therefore, it was expected that scores of the WFIRS-P would, at the minimum, correlate moderately (*r* > 0.30) [54] with the ADHD-RS-IV total score as well as the Inattention and Hyperactivity/Impulsivity subscales. The CGI–S is a severity rating of mental illness. It has been shown that mental health conditions in children can/may have larger negative effects on test scores, school attainment and function in general than physical health conditions [55]. Therefore, it was expected that all domains and the summary index of the WFIRS-P would at the minimum correlate moderately (*r* > 0.30) with the CGI–S data.

The responsiveness of a PRO instrument is deemed to be an important measurement property in order for the instrument to be considered a valid endpoint in clinical trials (reference PRO Guidance Document for Industry) [44]. The responsiveness of a PRO instrument is best determined by evaluating the instrument’s ability to detect true changes in the underlying condition being studied or treated. The ability of the WFIRS-P to detect clinically important changes over time [56] was assessed using the method of known-groups validity [57]. The criterion measure for this purpose was the treatment responder definition used in each trial wherein patients were classified as a responder if their ADHD-RS-IV total score improved by ≥30 % from baseline to the follow-up assessment *and* the CGI–I ratings were at least 1 (‘very much improved’) or 2 (‘much improved’) at the follow-up assessment. Student’s *t*-tests were conducted to test the statistical significance of the difference in mean WFIRS-P change scores between responders and non-responders. It was hypothesized that responders would show statistically significantly larger (*p <* 0.05) improvement on the WFIRS-P domain and summary index scores than non-responders.

An analysis of differential item functioning (DIF) was conducted using logistic regression methods to determine whether the items of each WFIRS-P scale showed any measurement bias between children (6–12 years) and adolescents (13–17 years) [58]. The results and interpretation of the DIF analyses are presented in Additional file 1.

## Results

Table 1 provides a brief description of each of the seven trials used for this study. Baseline demographic characteristics for the two random half-samples of study participants (sample 1: *n* *=* 1185; sample 2: *n* *=* 1172), along with scores for WFIRS-P and clinical measures, are shown in Table 2. Mean (standard deviation) ages were 11.0 (2.9) and 11.1 (2.9) years in samples 1 and 2, respectively, and approximately two-thirds of participants were children aged 6–12 years. The majority of participants were male (~75 %). Mean baseline scores for each clinical assessment and the six WFIRS-P domains were nearly identical between the two random half-samples.

**Table 2:** Characteristics of the study population at baseline

Characteristic	Sample 1^a^	Sample 2^b^
*n*	1185	1172
Demographics		
Age, years, mean (SD)	11.0 (2.9)	11.1 (2.9)
Age 6–12 years, n (%)	793 (67)	782 (67)
Age 13–17 years, n (%)	392 (33)	390 (33)
Gender, n (%)		
Female	292 (25)	316 (27)
Male	893 (75)	856 (73)
Clinical measures, mean (SD)		
ADHD-RS-IV		
Total scale	42.0 (6.5)	42.0 (6.5)
Inattention	19.3 (5.3)	19.3 (5.3)
Hyperactivity Impulsivity	22.7 (3.2)	22.6 (3.4)
CGI–S	4.9 (0.8)	4.9 (0.8)
WFIRS-P, mean (SD)		
Family domain	1.24 (0.78)	1.24 (0.76)
Learning and School domain	1.31 (0.65)	1.28 (0.64)
Life Skills domain	1.14 (0.53)	1.11 (0.51)
Child’s Self-Concept domain	0.91 (0.79)	0.90 (0.78)
Social Activities domain	0.96 (0.73)	0.95 (0.71)
Risky Activities domain	0.48 (0.40)	0.46 (0.38)
WFIRS-P summary index	1.03 (0.47)	1.01 (0.45)

^a^Sample 1 refers to the first random split half-sample from the pooled clinical trial data. ^b^Sample 2 is the second random split half-sample

*ADHD-RS-IV*ADHD Rating Scale Version IV, *CGI–S* Clinical Global Impression–Severity, *SD* standard deviation, *WFIRS-P* Weiss Functional Impairment Rating Scale-Parent Report Form

### Conceptual model

Overall fit of the six-domain model representing the conceptual framework of the WFIRS-P as measured by CFI was close to, but did not reach, the minimum threshold (>0.90) for acceptable model fit (Table 3; Additional file 2). CFI was 0.789 (sample 1) and 0.818 (sample 2) at baseline and 0.861 (sample 1) and 0.880 (sample 2) at follow-up assessments. The other indicator of model fit, RMSEA, was within the range of acceptable model fit (<0.10) for the six-domain model, ranging from 0.084 to 0.094 across analyses conducted at baseline and follow-up assessments. With few exceptions, item factor loadings for the six-factor model exceeded the expected magnitude for item convergence (*r* > 0.40). The exceptions occurred for the Life Skills domain items (excessive use of TV, computer or video games and keeping clean, brushing teeth, brushing hair, bathing, etc.) and the Risky Activities domain items (smoking cigarettes and taking illegal drugs). By comparison, the one-factor models showed poorer model fit as indicated by both CFI and RMSEA fit statistics. The CFI for a one-factor model ranged from 0.545 to 0.710 and RMSEA ranged from 0.133 to 0.141 across analyses. In addition, all item factor loadings were lower in the one-factor model compared with the six-factor model, and many items failed to show acceptable item convergence (*r* > 0.40) in the one-factor model.

**Table 3:** CFA of the WFIRS-P: item-to-factor loadings and fit statistics for one- and six-factor models

	Six-factor model				One-factor model			
	Baseline		Follow-up		Baseline		Follow-up	
	Sample 1	Sample 2	Sample 1	Sample 2	Sample 1	Sample 2	Sample 1	Sample 2
Range of factor loadings								
Family domain	0.671–0.868	0.612–0.881	0.676–0.896	0.655–0.892	0.611–0.814	0.552–0.818	0.616–0.839	0.595–0.837
Learning and School domain	0.485–0.911	0.449–0.890	0.585–0.900	0.621–0.927	0.347–0.718	0.326–0.713	0.470–0.733	0.512–0.773
Life Skills domain	0.277–0.722	0.268–0.721	0.372–0.730	0.338–0.754	0.199–0.549	0.182–0.492	0.299–0.583	0.272–0.609
Child’s Self-Concept domain	0.789–0.895	0.802–0.862	0.820–0.915	0.859–0.901	0.553–0.623	0.527–0.578	0.589–0.665	0.677–0.735
Social Activities domain	0.428–0.893	0.461–0.878	0.553–0.907	0.577–0.888	0.365–0.788	0.389–0.768	0.473–0.805	0.491–0.790
Risky Activities domain	0.287–0.808	0.289–0.790	0.356–0.858	0.929–0.818	0.200–0.670	0.206–0.623	0.284–0.714	0.930–0.672
Fit statistics								
CFI	0.789	0.818	0.861	0.880	0.545	0.579	0.665	0.710
RMSEA	0.094	0.092	0.084	0.085	0.141	0.141	0.134	0.133
TLI	0.929	0.922	0.951	0.954	0.840	0.817	0.875	0.888
Chi-Square	3099.9*	2730.8*	1544.1*	1534.1*	6360.1*	5997.1*	3415.9*	3411.8
df	270	253	206	195	259	248	196	192

*CFA*confirmatory factor analysis, *CFI*comparative fit index, *df* degrees of freedom, *RMSEA* root mean square error of approximation, *TLI*, Tucker Lewis Index, *WFIRS-P* Weiss Functional Impairment Rating Scale-Parent Report Form

*p < 0.001

### Reliability

All of the scales of the WFIRS-P demonstrated good internal consistency reliability (Additional file 3). Cronbach’s alpha exceeded 0.8 for all scales in the four samples except for Life Skills and Risky Activities, where values still exceeded the generally accepted cut-off of 0.7 [59]. A small subsample (sample 1: *n* *=* 35; sample 2: *n* *=* 34) met the criteria for a stable sample required to assess test–retest reliability as previously described. The ICCs observed for each scale met or exceeded acceptable test–retest reliability (*r* > 0.7). In sample 1, ICCs ranged between 0.73 and 0.89 across the six domains and summary index, with the exception of the Risky Activities domain where the ICC was 0.57. Similar results were found in sample 2.

### Convergent validity

At baseline, correlations between the WFIRS-P domains and summary index and the ADHD-RS-IV total score and Inattention and Hyperactivity/Impulsivity subscale scores and the CGI-S score ranged from near zero to moderate (maximum correlation = 0.39) (Table 4A). The Family domain and summary index showed the strongest correlations with the ADHD-RS-IV total score and Inattention subscale score in both samples 1 and 2, and the Learning and School and Life Skills domains and summary index showed the highest correlations with the ADHD-RS-IV Hyperactivity/Impulsivity subscale. The Family and Social Activities domains and the summary index showed the highest correlation with CGI-I in both samples 1 and 2. At follow-up, correlations between the WFIRS-P domains and summary index and the ADHD-RS-IV scales and CGI–S score were considerably stronger than those observed at baseline, and in most instances the correlations were moderate in strength as hypothesized (Table 4B). In samples 1 and 2, the Family and Learning and School domains and summary index showed the strongest correlations with the ADHD-RS-IV total score and both Inattention and Hyperactivity/Impulsivity subscales. Similarly, these WFIRS-P domains and summary index showed the strongest correlation with CGI–I in both samples 1 and 2.

**Table 4:** Relationship between WFIRS-P domain scores and summary index and ADHD criterion measures (ADHD-RS and CGI–S)

A. Baseline								
	Sample 1				Sample 2			
	ADHD-RS-IV			CGI–S	ADHD-RS-IV			CGI–S
	Total	Inattention	Hyperactivity Impulsivity		Total	Inattention	Hyperactivity Impulsivity	
Scale	rho	rho	rho	rho	rho	rho	rho	rho
Family domain	0.37	0.35	0.17	0.29	0.34	0.32	0.10	0.25
Learning and School domain	0.26	0.21	0.21	0.17	0.31	0.21	0.22	0.16
Life Skills domain	0.25	0.22	0.21	0.15	0.21	0.12	0.20	0.10
Child’s Self-Concept domain	0.11	0.07	0.11	0.15	0.02^ns^	0.01^ns^	0.07^a^	0.07
Social Activities domain	0.28	0.29	0.09	0.23	0.26	0.25	0.11	0.23
Risky Activities domain	0.25	0.28	0.10	0.19	0.28	0.26	0.10	0.18
WFIRS-P summary index	0.38	0.35	0.20	0.26	0.35	0.31	0.20	0.24
B. Follow-up								
	Sample 1				Sample 2			
	ADHD-RS-IV			CGI-S	ADHD-RS-IV			CGI-S
	Total	Inattention	Hyperactivity Impulsivity		Total	Inattention	Hyperactivity Impulsivity	
Scale	rho	rho	rho	rho	rho	rho	rho	rho
Family domain	0.45	0.42	0.41	0.36	0.47	0.46	0.41	0.43
Learning and School domain	0.52	0.41	0.50	0.41	0.50	0.44	0.47	0.44
Life Skills domain	0.41	0.33	0.41	0.34	0.36	0.33	0.38	0.34
Child’s Self-Concept domain	0.26	0.20	0.24	0.19	0.25	0.20	0.26	0.26
Social Activities domain	0.39	0.37	0.33	0.27	0.37	0.36	0.32	0.31
Risky Activities domain	0.35	0.37	0.26	0.29	0.41	0.41	0.33	0.35
WFIRS-P summary index	0.55	0.49	0.51	0.43	0.53	0.50	0.48	0.48

All correlations are statistically significant with *p <* 0.001 unless noted otherwise; ^a^p *<* 0.05; ^ns^ = not significant

*ADHD* attention-deficit/hyperactivity disorder, *ADHD-RS-IV* ADHD Rating Scale Version IV, *CGI–S* Clinical Global Impression–Severity, *r* Pearson correlation coefficient, *rho*Spearman rank correlation, *WFIRS-P* Weiss Functional Impairment Rating Scale-Parent Report Form

### Responsiveness

Approximately 70 % of patients were categorized as responders in both samples 1 and 2 (Table 5). As hypothesized, greater improvement (i.e. larger negative change scores) was found in the responder than the non-responder group across all WFIRS-P domains and the summary index, and these differences were statistically significant (*p* *<* 0.001). In the responder group, the largest improvement was observed for the Learning and School domain (−0.63 and −0.60 in samples 1 and 2, respectively), followed by the Family domain (−0.48 and −0.51 in samples 1 and 2, respectively). The Risky Activities domain had the smallest improvement among responders (−0.22 in both samples), but baseline scores were also the lowest for this domain, indicating less impairment to start with. Overall, change scores in the non-responder group were small, ranging from −0.09 for the Risky Activities domain in sample 2, to −0.21 for the Learning and School domain in sample 1.

**Table 5:** Comparison of mean change in WFIRS-P domain scores and summary index between responders and non-responders

	Sample 1				Sample 2			
	Responder^a^	Non-responder	T-statistic	*p*-value	Responder^a^	Non-responder	T-statistic	*p*-value
Scale	(*n* = 647)	(*n* = 277)			(*n* = 644)	(*n* = 300)		
Family domain	−0.48 (−0.6)	−0.20 (−0.5)	6.5	<0.0001	−0.51 (−0.6)	−0.16 (−0.5)	8.2	<0.0001
Learning and School domain	−0.63 (−0.6)	−0.21 (−0.5)	9.6	<0.0001	−0.60 (−0.6)	−0.15 (−0.6)	10.5	<0.0001
Life Skills domain	−0.38 (−0.5)	−0.17 (−0.4)	5.9	<0.0001	−0.39 (−0.5)	−0.19 (−0.5)	5.8	<0.0001
Child’s Self-Concept domain	−0.37 (−0.7)	−0.19 (−0.7)	3.5	0.0003	−0.34 (−0.7)	−0.09 (−0.7)	5.0	<0.0001
Social Activities domain	−0.38 (−0.6)	−0.18 (−0.5)	5.1	<0.0001	−0.38 (−0.6)	−0.13 (−0.6)	6.2	<0.0001
Risky Activities domain	−0.22 (−0.3)	−0.12 (−0.3)	4.2	<0.0001	−0.22 (−0.3)	−0.09 (−0.3)	5.7	<0.0001
WFIRS-P summary index	−0.42 (−0.4)	−0.17 (−0.3)	8.9	<0.0001	−0.42 (−0.4)	−0.15 (−0.3)	9.8	<0.0001

^a^Individuals were classified as a responder if their ADHD-RS-IV total score improved by 30 % or more from baseline to the follow-up assessment and the value on the CGI-I was at least a 1 (’very much improved’) or 2 (‘much improved’) at the follow-up assessment

*ADHD-RS-IV*ADHD Rating Scale Version IV, *CGI–I* Clinical Global Impression–Improvement, *WFIRS-P* Weiss Functional Impairment Rating Scale-Parent Report Form

## Discussion

The purpose of this study was to examine the reliability, validity and responsiveness of the WFIRS-P and to evaluate its appropriateness for assessing functional impairment in children and adolescents with ADHD in the context of clinical trials. To meet this objective, this study was conducted using pooled data from seven randomized controlled clinical trials designed to evaluate the safety/tolerability and efficacy of ADHD treatment. Overall, the results of this study support the reliability, validity and responsiveness of the WFIRS-P. The six-domain conceptual framework of the WFIRS-P as defined by the scoring algorithms showed adequate fit with CFA. Domain scores satisfied accepted minimum standards for internal consistency and test–retest reliability (*r* ≥ 0.7) [59]. Many of the WFIRS-P domains correlated significantly with the ADHD-RS-IV scales and CGI–S score, although to varying degrees. The WFIRS-P domain scores were also responsive to change. Mean changes in WFIRS-P domain scores differed significantly between responder and non-responder groups, with responders showing greater improvement in scores than non-responders. All results were replicated between two random samples, indicating that the WFIRS-P has robust psychometric measurement properties.

In evaluating these measurement properties, there were a few notable findings worthy of further discussion. First, while the CFI statistics did not reach pre-specified levels for model fit using CFA, model fit as determined by RMSEA was satisfactory and the tests of the six-domain conceptual framework of the WFIRS-P showed a much better model fit compared with a one-factor model. Fit statistics (CFI and RMSEA) for the six-factor model were much better than those observed in the one-factor model, and item-to-factor loadings in the six-factor model were all stronger than those observed in the one-factor model. Furthermore, in the one-factor model, many items showed item-factor loadings of <0.4, which is a minimum standard for item-convergent validity [49] calling into question the interpretation of a single global score using item-level data. In an effort to improve model fit, CFA analyses were conducted with an alternative specification of the conceptual framework of the WFIRS-P. Model fit was shown to improve considerably (CFI > 0.9 and RMSEA < 0.1) when the items of the School Learning-Behavior scale were modelled as two concepts, School Learning and School Behavior. Future studies should focus on testing these two concepts separately using other sources of WFIRS-P data, such as observational study data.

In this study, the availability of criterion measures to evaluate the validity of the WFIRS-P domains was limited to ADHD symptoms, as measured by the ADHD-RS-IV scale, and ADHD severity, as measured by the CGI–S scale. In order to demonstrate convergent validity, it is generally recommended that the correlation between the measure in question (WFIRS-P) and the criterion measure meet or exceed 0.30 [60]. However, given the lack of studies conducted that have investigated the measurement properties of the WFIRS-P, there was little basis to formulate hypotheses about the magnitude of correlation that should be observed between the WFIRS-P domains and the ADHD-RS-IV and CGI–S scales in this study. Hypotheses were generated under the general framework that more symptoms (frequency or severity) or greater severity should be associated with greater functional impairment [61]. In this study, it was found that some concepts of the WFIRS-P showed higher correlations with symptoms (ADHD-RS-IV) and severity (CGI–S) of ADHD than others. For example, the Family, Learning and School, and Life Skills domains generally showed stronger correlations with ADHD-RS-IV and CGI–S than other WFIRS-P domains such as Child Self-Concept or Risky Activities. These findings do not necessarily invalidate those WFIRS-P domains with lower correlations, but rather help us to understand what concepts are more proximal to, and what concepts appear to be more distal to, the symptoms and severity of ADHD. This finding is consistent with results of qualitative studies conducted to define a measurement model for assessing functional impairment in ADHD [62]. One implication of this finding concerns selecting specific WFIRS-P domains for evaluating treatment efficacy. If treatment is aimed at reducing the symptoms and severity of ADHD, then it is more likely the case that domains showing stronger correlations with ADHD symptoms and severity will respond to treatment than domains showing weaker correlations with ADHD symptoms and severity. More importantly, these results reinforce the need for further exploration of the validity of the WFIRS-P using criterion measures other than those related to the symptoms and severity of ADHD, in particular criterion measures that are patient-based or parent-based as opposed to clinician-based, as they may have differing perspectives on patient functional impairment.

Another observation in this study highlights that the results of analyses conducted with follow-up data yielded better measurement properties than those conducted with baseline data. For example, model fit statistics from CFA were much better with follow-up data, and correlations between WFIRS-P domain scores and the criterion measures were considerably stronger with follow-up data. This may have been due in part to the impact of the inclusion and exclusion criteria of each study on baseline data. The inclusion and exclusion criteria of each study were in part designed to identify patients with more severe symptoms of ADHD, which resulted in a fairly homogenous sample with respect to symptoms, severity and functional impairment. In fact, the inclusion and exclusion criteria in all seven studies included a minimum ADHD-RS-IV total score, and the four GXR trials also included a minimum CGI–S score, both of which were criterion measures used to evaluate the convergent validity of the WFIRS-P. As a consequence, there was less variability in the criterion measures at baseline, resulting in attenuated correlations between the WFIRS-P and the criterion measures. At follow-up, however, study inclusion and exclusion criteria were likely to be less impactful as half of the patients of each trial were treated and thus were expected to improve in symptoms and severity versus those in the placebo group. As a consequence, the sample at follow-up was more heterogeneous with respect to underlying symptoms, severity and functional impairment, all of which contributed to the better psychometric results observed with follow-up data.

Several limitations of this study should be considered when interpreting the study results. First, the study population was enrolled into randomized controlled clinical trials using stringent inclusion and exclusion criteria. This population may not be representative of all children and adolescents with ADHD seen in general practice settings, and hence it is not known how well the WFIRS-P would perform in a more general patient population from these study results. However, in determining the adequacy of an instrument for measuring functional impairment in clinical trials of ADHD treatment, the results of this study suggest that the WFIRS-P has acceptable measurement properties that support the reliability and validity of the instrument as a measure of functional impairment in clinical trials.

Another potential limitation of this study concerns the limited number of criterion measures available across all trials for purposes of assessing the convergent-divergent validity and responsiveness of the WFIRS-P domains. The criterion measures relied upon in this study were symptom-based and severity measures were clinician-reported. While it was expected that more severe symptoms would be associated with greater functional impairment, the results of this study showed that some domains of the WFIRS-P were weakly correlated with the criterion measures and that correlations tended to be low in general. For example, the Child’s Self-Concept domain was weakly correlated with the ADHD-RS-IV. This finding does not necessarily invalidate the Child’s Self-Concept domain, but calls into question the appropriateness of the ADHD-RS-IV as a criterion measure for validating this domain. It has been reported that some concepts of ADHD-related functional impairment are more distal to the symptoms of ADHD than others, which would help explain the low correlations observed for some WFIRS-P domains [62].

The fact that the criterion measures were clinician reported may also factor into the interpretation of the convergent validity correlations observed in this study. While for many WFIRS-P domains correlations with the criterion measures met the minimum threshold (*r* > 0.3) for convergent validity, the WFIRS-P Child’s Self-Concept domain showed lower correlations than the minimum threshold. This may be driven in part by the use of clinician-reported measures as criterion measures, which do not always reflect the patients’ or parents’ perspectives. Further study of the validity of the WFIRS-P domains would benefit from the use of other conceptually related patient-reported outcome measures.

## Conclusion

Despite the limitations of this study, the evidence generated from the analyses conducted showed adequate support for the six-domain conceptual framework of the WFIRS-P and demonstrated that the WFIRS-P domains are reliable, valid and responsive. The evidence supporting the conceptual framework of the WFIRS-P items and domains was adequate and replicated between random half-samples. All WFIRS-P domains met the minimum standards of internal consistency reliability and test–retest reliability, and with few exceptions, correlations between WFIRS-P domains and each criterion measure met the minimum value to support convergent validity. Lastly, all WFIRS-P domains were shown to be responsive to changes in ADHD status as defined using criteria implemented to determine treatment response. These findings support the use of the WFIRS-P as a measure of functional impairment in clinical trials of children and adolescents with ADHD.

## Additional files

Additional file 1:
**Differential item functioning test of** **WFIRS-P.** (DOCX 33 kb) Additional file 2:
**Confirmatory factor analysis of the WFIRS-P factor loadings for the six-factor and one-factor models.** (DOCX 27 kb) Additional file 3:
**Internal consistency and test–retest reliability of the WFIRS-P.** (DOCX 19 kb)
