# Supplementary material for: Psychometric validation of the Weiss Functional Impairment Rating Scale-Parent Report Form in children and adolescents with attention-deficit/hyperactivity disorder
Source: Health Qual Life Outcomes. 2015 Nov 17;13:184. doi: 10.1186/s12955-015-0379-1 (PMC4650258; doi:10.1186/s12955-015-0379-1)
Supplement: Additional file 1: — Differential item functioning test of WFIRS-P. (DOCX 33 kb) [file 12955_2015_379_MOESM1_ESM.docx]

**Differential item functioning test of WFIRS-P**

**Methods**

Differential item functioning (DIF) occurs when respondents of different groups endorse questionnaire items differently, given that respondents are roughly equal in terms of the underlying latent trait being measured. A logistic regression approach was used to test for the presence of uniform and non-uniform DIF in each Weiss Functional Impairment Rating Scale-Parent Report Form (WFIRS-P) domain item between children (6–12 years) and adolescents (13–17 years) (Zumbo, 1999). In this context, DIF was assessed by testing the association between each WFIRS-P item in a domain and age group (6–12 vs 13–17 years) while conditioning on the aggregate sum score for each WFIRS-P domain score. The magnitude of DIF was quantified by a pseudo R^2^ difference measure that expresses the increase in explained item-response variance due to age group differences. For the purposes of this study, an increase in variance by 2% or more was considered an indication of DIF between age groups (Zumbo, 1999). DIF tests were conducted at baseline and follow-up visits using the pooled sample of patients from the seven clinical trials.

**Results**

Results showed that significant DIF between age groups was not present in five of six domains, and was only found in the Risky Activities domain of the WFIRS-P. Five of the ten items of the Risky Activities domain were found to have significant DIF between the two age groups. These items included ‘doing things that are illegal’, ‘being involved with the police’, smoking cigarettes’, ‘taking illegal drugs’ and ‘sexually inappropriate behaviour’. All other items/domains showed no significant DIF between the two age groups.

**Discussion**

Overall, five of the domains of the WFIRS-P showed no DIF between the two age groups tested, representing children (6–12 years) and adolescents (13–17 years). Scores for the WFIRS-P Family, Learning and School, Life Skills, Child’s Self-Concept, and Social domains have similar meaning and interpretation for both children and adolescents. However, half of the items of the WFIRS-P Risky Activities domain showed significant DIF between children and adolescents, meaning that scores from this domain do not have the same meaning or interpretation for these two age groups. The results of the DIF analyses for the WFIRS-P Risky Activities domain make sense from a practical standpoint. Children aged 12 years and under are not likely to engage in the activities found to have DIF in the Risky Activities domains (‘doing things that are illegal’, ‘being involved with the police’, ‘smoking cigarettes’, ‘taking illegal drugs’ and ‘sexually inappropriate behaviour’). On the other hand, it is plausible that children aged 12 years and under could engage in activities represented in the other five items of the WFIRS-P Risky Activities domain that showed no DIF (‘easily led by other children’, ‘breaking or damaging things’, ‘doing dangerous things’, ‘causes injury to others’ and ‘says mean or inappropriate things’).

**Reference**

1. Zumbo BD. A handbook on the theory and methods of differential item functioning (DIF): logistic regression modeling as a unitary framework for binary and Likert-type (ordinal) item scores. Ottawa,ON: Directorate of Human Resources Research and Evaluation, Department of National Defense; 1999.

Differential item functioning analysis of WFIRS-P Risky Behaviour items by age group (6–12 vs 13–17 years)

| Item | Item label | ChiSq1 | df1 | Prob1 | ChiSq2 | df2 | Prob2 | ChiSq3 | df3 | Prob3 | R^2^DIF |
| --- | --- | --- | --- | --- | --- | --- | --- | --- | --- | --- | --- |
| *Baseline sample (n* *= 2357)* | | | | | | | | | | | |
| WFIRSF01 | Easily led by other children | 4.41482 | 1 | 0.03563 | 0.01041 | 1 | 0.99481 | 4.42523 | 2 | 0.10941 | 0.00138 |
| WFIRSF02 | Breaking or damaging things | 14.98434 | 1 | 0.00011 | 0.00979 | 1 | 0.99512 | 14.99413 | 2 | 0.00055 | 0.00349 |
| **WFIRSF03** | **Doing things that are illegal** | **58.65817** | **1** | **0.00000** | **0.13159** | **1** | **0.93632** | **58.78976** | **2** | **0.00000** | **0.03362** |
| **WFIRSF04** | **Being involved with the police** | **59.39938** | **1** | **0.00000** | **0.31356** | **1** | **0.85489** | **59.71294** | **2** | **0.00000** | **0.06545** |
| **WFIRSF05** | **Smoking cigarettes** | **160.56868** | **1** | **0.00000** | **0.20882** | **1** | **0.90085** | **160.77750** | **2** | **0.00000** | **0.18113** |
| **WFIRSF06** | **Taking illegal drugs** | **68.29660** | **1** | **0.00000** | **2.11005** | **1** | **0.34818** | **70.40665** | **2** | **0.00000** | **0.20929** |
| WFIRSF07 | Doing dangerous things | 0.80193 | 1 | 0.37052 | 1.71457 | 1 | 0.42431 | 2.51650 | 2 | 0.28415 | 0.00095 |
| WFIRSF08 | Causes injury to others | 21.91170 | 1 | 0.00000 | 0.84344 | 1 | 0.65592 | 22.75514 | 2 | 0.00001 | 0.00880 |
| WFIRSF09 | Says mean or inappropriate things | 7.61095 | 1 | 0.00580 | 4.11100 | 1 | 0.12803 | 11.72195 | 2 | 0.00285 | 0.00340 |
| **WFIRSF10** | **Sexually inappropriate behaviour** | **34.39907** | **1** | **0.00000** | **1.15048** | **1** | **0.56257** | **35.54954** | **2** | **0.00000** | **0.02655** |
| *Follow-up sample (n = 1912)* | | | | | | | | | | | |
| WFIRSF01 | Easily led by other children | 1.27564 | 1 | 0.25871 | 0.05398 | 1 | 0.97337 | 1.32962 | 2 | 0.51437 | 0.00065 |
| WFIRSF02 | Breaking or damaging things | 4.89710 | 1 | 0.02690 | 0.00285 | 1 | 0.99858 | 4.89995 | 2 | 0.08630 | 0.00174 |
| **WFIRSF03** | **Doing things that are illegal** | **28.60749** | **1** | **0.00000** | **0.13567** | **1** | **0.93441** | **28.74316** | **2** | **0.00000** | **0.03445** |
| **WFIRSF04** | **Being involved with the police** | **36.90927** | **1** | **0.00000** | **0.01343** | **1** | **0.99331** | **36.92270** | **2** | **0.00000** | **0.09639** |
| **WFIRSF05** | **Smoking cigarettes** | **109.93090** | **1** | **0.00000** | **1.08191** | **1** | **0.58219** | **111.01281** | **2** | **0.00000** | **0.19250** |
| **WFIRSF06** | **Taking illegal drugs** | **20.38168** | **1** | **0.00001** | **0.04256** | **1** | **0.97894** | **20.42425** | **2** | **0.00004** | **0.15359** |
| WFIRSF07 | Doing dangerous things | 3.05145 | 1 | 0.08066 | 0.43860 | 1 | 0.80308 | 3.49005 | 2 | 0.17464 | 0.00230 |
| WFIRSF08 | Causes injury to others | 2.08092 | 1 | 0.14915 | 0.88303 | 1 | 0.64306 | 2.96394 | 2 | 0.22719 | 0.00226 |
| WFIRSF09 | Says mean or inappropriate things | 1.90689 | 1 | 0.16731 | 0.24895 | 1 | 0.88296 | 2.15583 | 2 | 0.34030 | 0.00099 |
| **WFIRSF10** | **Sexually inappropriate behaviour** | **22.19021** | **1** | **0.00000** | **2.06121** | **1** | **0.35679** | **24.25142** | **2** | **0.00001** | **0.03492** |

Note: Items in bolded text were found to have significant DIF.

DIF, differential item functioning; WFIRS-P, Weiss Functional Impairment Rating Scale-Parent Report Form.
