# Supplementary material for: Psychometric validation of the Weiss Functional Impairment Rating Scale-Parent Report Form in children and adolescents with attention-deficit/hyperactivity disorder
Source: Health Qual Life Outcomes. 2015 Nov 17;13:184. doi: 10.1186/s12955-015-0379-1 (PMC4650258; doi:10.1186/s12955-015-0379-1)
Supplement: Additional file 2: — Confirmatory factor analysis of the WFIRS-P factor loadings for the six-factor and one-factor models. (DOCX 27 kb) [file 12955_2015_379_MOESM2_ESM.docx]

**Confirmatory factor analysis of the WFIRS-P factor loadings for the six-factor and one-factor models**

|  | Six-factor model | | | | One-factor model | | | |
| --- | --- | --- | --- | --- | --- | --- | --- | --- |
|  | Baseline | | Follow-up | | Baseline | | Follow-up | |
|  | Sample 1 | Sample 2 | Sample 1 | Sample 2 | Sample 1 | Sample 2 | Sample 1 | Sample 2 |
| **Family** |  |  |  |  |  |  |  |  |
| Problems with siblings | 0.671 (0.020) | 0.612 (0.023) | 0.676 (0.025) | 0.655 (0.024) | 0.611 (0.020) | 0.552 (0.023) | 0.616 (0.024) | 0.595 (0.024) |
| Problems between parents | 0.754 (0.016) | 0.751 (0.017) | 0.758 (0.019) | 0.753 (0.020) | 0.694 (0.017) | 0.683 (0.017) | 0.697 (0.020) | 0.695 (0.020) |
| Takes time away from family | 0.741 (0.016) | 0.771 (0.015) | 0.794 (0.017) | 0.824 (0.014) | 0.682 (0.016) | 0.706 (0.016) | 0.731 (0.017) | 0.767 (0.015) |
| Causing fighting in the family | 0.841 (0.012) | 0.815 (0.013) | 0.823 (0.015) | 0.844 (0.014) | 0.783 (0.013) | 0.749 (0.014) | 0.766 (0.016) | 0.787 (0.015) |
| Isolating the family | 0.817 (0.016) | 0.796 (0.018) | 0.841 (0.018) | 0.835 (0.018) | 0.754 (0.016) | 0.726 (0.018) | 0.774 (0.018) | 0.772 (0.018) |
| Hard for family to have fun | 0.868 (0.010) | 0.881 (0.010) | 0.879 (0.013) | 0.859 (0.013) | 0.813 (0.011) | 0.818 (0.011) | 0.819 (0.014) | 0.805 (0.014) |
| Makes parenting difficult | 0.846 (0.012) | 0.823 (0.013) | 0.858 (0.013) | 0.852 (0.012) | 0.795 (0.012) | 0.763 (0.013) | 0.803 (0.014) | 0.794 (0.013) |
| Hard to give fair attention | 0.867 (0.011) | 0.842 (0.012) | 0.896 (0.011) | 0.892 (0.011) | 0.814 (0.011) | 0.783 (0.012) | 0.839 (0.012) | 0.837 (0.012) |
| Provokes others to hit/scream | 0.788 (0.015) | 0.730 (0.018) | 0.785 (0.018) | 0.738 (0.020) | 0.725 (0.015) | 0.662 (0.018) | 0.719 (0.018) | 0.676 (0.020) |
| Costs family more money | 0.696 (0.020) | 0.702 (0.021) | 0.713 (0.024) | 0.722 (0.023) | 0.634 (0.020) | 0.636 (0.021) | 0.650 (0.023) | 0.662 (0.023) |
| **Learning and School** |  |  |  |  |  |  |  |  |
| Difficulty keeping up with schoolwork | 0.618 (0.023) | 0.673 (0.021) | 0.780 (0.017) | 0.812 (0.016) | 0.436 (0.025) | 0.521 (0.023) | 0.643 (0.020) | 0.690 (0.018) |
| Needs extra help at school | 0.718 (0.018) | 0.798 (0.015) | 0.852 (0.013) | 0.851 (0.014) | 0.488 (0.023) | 0.613 (0.019) | 0.721 (0.016) | 0.732 (0.017) |
| Needs tutoring | 0.645 (0.021) | 0.747 (0.016) | 0.787 (0.016) | 0.753 (0.018) | 0.434 (0.024) | 0.570 (0.020) | 0.667 (0.019) | 0.640 (0.020) |
| Grades not as good as ability | 0.485 (0.028) | 0.449 (0.029) | 0.609 (0.025) | 0.639 (0.025) | 0.347 (0.027) | 0.326 (0.027) | 0.482 (0.026) | 0.526 (0.025) |
| Time-out/ removal from class | 0.814 (0.016) | 0.807 (0.017) | 0.855 (0.018) | 0.820 (0.018) | 0.649 (0.018) | 0.650 (0.018) | 0.701 (0.019) | 0.694 (0.019) |
| Problems in the school yard | 0.806 (0.016) | 0.796 (0.016) | 0.782 (0.021) | 0.813 (0.019) | 0.642 (0.019) | 0.639 (0.019) | 0.645 (0.023) | 0.691 (0.021) |
| Receives detentions | 0.911 (0.017) | 0.890 (0.017) | 0.900 (0.020) | 0.927 (0.018) | 0.718 (0.016) | 0.713 (0.016) | 0.733 (0.019) | 0.773 (0.017) |
| Suspended or expelled | 0.768 (0.021) | 0.730 (0.023) | 0.778 (0.029) | 0.795 (0.023) | 0.601 (0.023) | 0.576 (0.024) | 0.632 (0.029) | 0.671 (0.025) |
| Misses/late to classes | 0.688 (0.031) | 0.625 (0.032) | 0.609 (0.045) | 0.698 (0.037) | 0.525 (0.030) | 0.472 (0.031) | 0.489 (0.042) | 0.575 (0.037) |
| Causes problems for teacher | 0.501 (0.034) | 0.557 (0.033) | 0.585 (0.037) | 0.621 (0.035) | 0.384 (0.030) | 0.453 (0.028) | 0.470 (0.033) | 0.512 (0.031) |
| **Life Skills** |  |  |  |  |  |  |  |  |
| Excessive use of TV, etc. | 0.277 (0.035) | 0.353 (0.035) | 0.496 (0.033) | 0.495 (0.034) | 0.199 (0.029) | 0.238 (0.028) | 0.400 (0.029) | 0.400 (0.029) |
| Keeping clean, etc. | 0.317 (0.034) | 0.268 (0.037) | 0.372 (0.037) | 0.338 (0.038) | 0.236 (0.028) | 0.182 (0.029) | 0.299 (0.032) | 0.272 (0.032) |
| Problems getting ready for school | 0.645 (0.023) | 0.659 (0.024) | 0.703 (0.024) | 0.739 (0.021) | 0.478 (0.023) | 0.456 (0.024) | 0.565 (0.024) | 0.599 (0.022) |
| Problems getting ready for bed | 0.713 (0.022) | 0.721 (0.022) | 0.730 (0.021) | 0.754 (0.020) | 0.522 (0.022) | 0.492 (0.023) | 0.583 (0.023) | 0.609 (0.022) |
| Problems with eating | 0.475 (0.030) | 0.424 (0.033) | 0.500 (0.032) | 0.511 (0.033) | 0.349 (0.026) | 0.283 (0.028) | 0.399 (0.029) | 0.412 (0.029) |
| Problems with sleeping | 0.531 (0.030) | 0.520 (0.030) | 0.519 (0.032) | 0.590 (0.032) | 0.392 (0.026) | 0.354 (0.027) | 0.414 (0.029) | 0.477 (0.029) |
| Gets hurt or injured | 0.722 (0.029) | 0.620 (0.033) | 0.694 (0.033) | 0.700 (0.033) | 0.549 (0.023) | 0.448 (0.026) | 0.565 (0.028) | 0.570 (0.029) |
| Avoids exercise | 0.400 (0.037) | 0.421 (0.039) | 0.460 (0.038) | 0.534 (0.036) | 0.301 (0.030) | 0.296 (0.031) | 0.370 (0.034) | 0.434 (0.032) |
| Needs more medical care | 0.656 (0.032) | 0.652 (0.035) | 0.642 (0.037) | 0.690 (0.035) | 0.501 (0.027) | 0.473 (0.028) | 0.522 (0.032) | 0.563 (0.031) |
| Trouble taking medication, etc. | 0.454 (0.038) | 0.467 (0.038) | 0.528 (0.040) | 0.578 (0.039) | 0.344 (0.031) | 0.329 (0.031) | 0.426 (0.035) | 0.469 (0.034) |
| **Child’s Self-Concept** |  |  |  |  |  |  |  |  |
| Feels bad about self | 0.789 (0.018) | 0.833 (0.018) | 0.839 (0.019) | 0.899 (0.014) | 0.553 (0.021) | 0.556 (0.021) | 0.612 (0.023) | 0.725 (0.017) |
| Does not have enough fun | 0.804 (0.020) | 0.802 (0.022) | 0.820 (0.021) | 0.859 (0.019) | 0.558 (0.022) | 0.527 (0.024) | 0.589 (0.025) | 0.677 (0.021) |
| Not happy with life | 0.895 (0.016) | 0.862 (0.018) | 0.915 (0.017) | 0.901 (0.016) | 0.623 (0.020) | 0.578 (0.022) | 0.665 (0.023) | 0.735 (0.019) |
| **Social Activities** |  |  |  |  |  |  |  |  |
| Teased or bullied | 0.676 (0.021) | 0.659 (0.022) | 0.751 (0.022) | 0.674 (0.024) | 0.583 (0.021) | 0.558 (0.021) | 0.657 (0.022) | 0.579 (0.024) |
| Teases or bullies others | 0.781 (0.019) | 0.757 (0.022) | 0.827 (0.021) | 0.812 (0.022) | 0.678 (0.017) | 0.643 (0.019) | 0.717 (0.020) | 0.694 (0.021) |
| Problems getting along | 0.893 (0.010) | 0.863 (0.012) | 0.907 (0.012) | 0.882 (0.013) | 0.788 (0.012) | 0.751 (0.014) | 0.805 (0.014) | 0.773 (0.015) |
| Problems participating in activities | 0.428 (0.031) | 0.461 (0.030) | 0.553 (0.033) | 0.577 (0.033) | 0.365 (0.028) | 0.389 (0.027) | 0.473 (0.030) | 0.491 (0.030) |
| Problems making friends | 0.719 (0.019) | 0.696 (0.019) | 0.756 (0.020) | 0.754 (0.020) | 0.630 (0.020) | 0.595 (0.020) | 0.668 (0.022) | 0.660 (0.021) |
| Problems keeping friends | 0.872 (0.011) | 0.878 (0.011) | 0.862 (0.014) | 0.888 (0.012) | 0.776 (0.013) | 0.768 (0.013) | 0.769 (0.016) | 0.790 (0.015) |
| Trouble with parties | 0.830 (0.015) | 0.823 (0.016) | 0.829 (0.018) | 0.866 (0.017) | 0.730 (0.016) | 0.714 (0.017) | 0.727 (0.020) | 0.762 (0.018) |
| **Risky Activities** |  |  |  |  |  |  |  |  |
| Easily led by others | 0.656 (0.024) | 0.621 (0.027) | 0.730 (0.027) | 0.721 (0.026) | 0.542 (0.021) | 0.499 (0.023) | 0.607 (0.023) | 0.593 (0.023) |
| Breaking/damaging things | 0.801 (0.019) | 0.790 (0.020) | 0.858 (0.019) | 0.818 (0.022) | 0.655 (0.018) | 0.623 (0.019) | 0.714 (0.019) | 0.672 (0.021) |
| Doing illegal things | 0.682 (0.033) | 0.659 (0.038) | 0.687 (0.047) | 0.718 (0.045) | 0.555 (0.032) | 0.518 (0.035) | 0.570 (0.046) | 0.596 (0.041) |
| Involved with the police | 0.633 (0.052) | 0.673 (0.059) | 0.577 (0.079) | 0.686 (0.060) | 0.510 (0.048) | 0.526 (0.053) | 0.471 (0.078) | 0.582 (0.058) |
| Smoking cigarettes | 0.360 (0.061) | 0.494 (0.067) | 0.356 (0.065) | 0.371 (0.071) | 0.274 (0.054) | 0.382 (0.059) | 0.284 (0.057) | 0.305 (0.063) |
| Taking illegal drugs | 0.287 (0.097) | 0.289 (0.061) | 0.596 (0.115) | 0.549 (0.044) | 0.200 (0.082) | 0.206 (0.052) | 0.474 (0.107) | 0.430 (0.032) |
| Doing dangerous things | 0.661 (0.027) | 0.681 (0.029) | 0.750 (0.030) | 0.664 (0.035) | 0.544 (0.026) | 0.543 (0.027) | 0.634 (0.031) | 0.548 (0.032) |
| Causes injury to others | 0.808 (0.022) | 0.761 (0.028) | 0.793 (0.033) | 0.765 (0.035) | 0.670 (0.022) | 0.611 (0.026) | 0.669 (0.032) | 0.632 (0.033) |
| Says mean/inappropriate things | 0.766 (0.020) | 0.750 (0.022) | 0.761 (0.024) | 0.779 (0.024) | 0.627 (0.019) | 0.594 (0.020) | 0.629 (0.023) | 0.639 (0.022) |
| Sexually inappropriate behaviour | 0.589 (0.044) | 0.489 (0.049) | 0.591 (0.055) | 0.694 (0.055) | 0.487 (0.040) | 0.385 (0.042) | 0.488 (0.049) | 0.586 (0.051) |

WFIRS-P, Weiss Functional Impairment Rating Scale–Parent Report Form.
