# Supplementary material for: Psychometric validation of the Weiss Functional Impairment Rating Scale-Parent Report Form in children and adolescents with attention-deficit/hyperactivity disorder
Source: Health Qual Life Outcomes. 2015 Nov 17;13:184. doi: 10.1186/s12955-015-0379-1 (PMC4650258; doi:10.1186/s12955-015-0379-1)
Supplement: Additional file 3: — Internal consistency and test–retest reliability of the WFIRS-P. (DOCX 19 kb) [file 12955_2015_379_MOESM3_ESM.docx]

Internal consistency and test–retest reliability of the WFIRS-P

| A. Internal consistency reliability (Cronbach's alpha) | | | | |
| --- | --- | --- | --- | --- |
|  | Baseline | | Follow-up | |
| Domain | Sample 1 | Sample 2 | Sample 1 | Sample 2 |
|  | n = 1185 | n = 1172 | n = 940 | n = 972 |
| Family | 0.92 | 0.91 | 0.92 | 0.92 |
| Learning and School | 0.83 | 0.82 | 0.86 | 0.87 |
| Life Skills | 0.72 | 0.71 | 0.75 | 0.76 |
| Child's Self-Concept | 0.81 | 0.82 | 0.83 | 0.86 |
| Social Activities | 0.86 | 0.85 | 0.86 | 0.86 |
| Risky Activities | 0.79 | 0.77 | 0.78 | 0.75 |
| WFIRS-P summary index | 0.94 | 0.93 | 0.94 | 0.95 |
| B. Test–retest reliability (ICC) | | | | |
| Domain | Sample 1 | | Sample 2 | |
|  | n = 35 | | n = 34 | |
| Family | 0.82 | | 0.77 | |
| Learning and School | 0.84 | | 0.86 | |
| Life Skills | 0.86 | | 0.75 | |
| Child's Self-Concept | 0.73 | | 0.79 | |
| Social Activities | 0.89 | | 0.73 | |
| Risky Activities | 0.77 | | 0.57 | |
| WFIRS-P summary index | 0.88 | | 0.82 | |

ICC, intraclass correlation coefficient; WFIRS-P, Weiss Functional Impairment Rating Scale-Parent Report Form.
